# The function of phosphatidylinositol 5-phosphate 4-kinase γ (PI5P4Kγ) explored using a specific inhibitor that targets the PI5P-binding site

**DOI:** 10.1042/BJ20141333

**Published:** 2015-02-20

**Authors:** Jonathan H. Clarke, Maria-Luisa Giudici, John E. Burke, Roger L. Williams, David J. Maloney, Juan Marugan, Robin F. Irvine

**Affiliations:** *Department of Pharmacology, Tennis Court Road, Cambridge CB2 1PD, U.K.; †MRC Laboratory of Molecular Biology, Francis Crick Avenue, Cambridge CB2 0QH, U.K.; ‡National Center for Advancing Translational Sciences, 9800 Medical Center Drive, Rockville, MD 20850, U.S.A.

**Keywords:** phosphatidylinositol 5-phosphate (PI5P), phosphatidylinositol 5-phosphate 4-kinase (PI5P4K), phosphatidylinositol 5-phosphate 4-kinase γ (PI5P4Kγ), HDX, hydrogen–deuterium exchange, HDX-MS, hydrogen-deuterium exchange mass spectrometry, mpkCCD, mouse principal kidney cortical collecting duct, PI5P, phosphatidylinositol 5-phosphate, QT-PCR, quantitative PCR, TBP, TATA-box-binding protein, TCEP, tris carboxyl ethyl phosphine, UPLC, ultra performance liquid chromatography, WT, wild-type

## Abstract

NIH-12848 (NCGC00012848-02), a putative phosphatidylinositol 5-phosphate 4-kinase γ (PI5P4Kγ) inhibitor, was explored as a tool for investigating this enigmatic, low activity, lipid kinase. PI5P4K assays *in vitro* showed that NIH-12848 inhibited PI5P4Kγ with an IC_50_ of approximately 1 μM but did not inhibit the α and β PI5P4K isoforms at concentrations up to 100 μM. A lack of inhibition of PI5P4Kγ ATPase activity suggested that NIH-12848 does not interact with the enzyme's ATP-binding site and direct exploration of binding using hydrogen–deuterium exchange (HDX)-MS (HDX-MS) revealed the putative PI5P-binding site of PI5P4Kγ to be the likely region of interaction. This was confirmed by a series of mutation experiments which led to the identification of a single PI5P4Kγ amino acid residue that can be mutated to its PI5P4Ks α and β homologue to render PI5P4Kγ resistant NIH-12848 inhibition. NIH-12848 (10 μM) was applied to cultured mouse principal kidney cortical collecting duct (mpkCCD) cells which, we show, express PI5P4Kγ that increases when the cells grow to confluence and polarize. NIH-12848 inhibited the translocation of Na^+^/K^+^-ATPase to the plasma membrane that occurs when mpkCCD cells grow to confluence and also prevented reversibly their forming of ‘domes’ on the culture dish. Both these NIH-12848-induced effects were mimicked by specific RNAi knockdown of PI5P4Kγ, but not that of PI5P4Ks α or β. Overall, the data reveal a probable contribution of PI5P4Kγ to the development and maintenance of epithelial cell functional polarity and show that NIH-12848 is a potentially powerful tool for exploring the cell physiology of PI5P4Ks.

## Abstract 2

We have characterised a specific inhibitor of the enzyme Phosphatidylinositol 5-phosphate 4-kinase γ, including establishing where on the enzyme the inhibitor binds, and then applied this inhibitor to a kidney cell line to elucidate the intracellular functions of the enzyme.

## INTRODUCTION

The phosphatidylinositol 5-phosphate 4-kinases (PI5P4Ks) are a family consisting of three isoforms in mammals (α, β and γ), whose most probable function is currently believed to be the regulation of the levels of their substrate, PI5P [1,2]. Although the α and β isoforms have had some physiological and pathological functions assigned to them, including in gene regulation [3,4], stress responses [5], insulin signalling [6] and cancer [7,8], PI5P4Kγ is hardly understood at all. It is apparently ubiquitously expressed, but at very different levels between tissues, being particularly high in epithelial cells of the thick ascending limb of the kidney and collecting ducts cells [9] and in specific neurons in brain [10]. It apparently has a vesicular localization [9,10], may have a functional connection with Rho [11], shows a much lower enzymatic activity *in vitro* than the other PI5P4K isoforms [12] and can heterodimerize *in vitro* with PI5P4Kα [12] (note that extensive heterodimerization between PI5P4Ks α and β has been shown to occur *in vivo* [13,14]).

Specific inhibitors of enzymes can be useful tools in studying their function and kinase inhibitors are among those that have shown most promise as potential therapeutic agents. Recently, the characterization of inhibitors for PI5P4Kα [15] and PI5P4Kβ [16] have raised that hope for those isoforms, but the isoform specificity of neither inhibitor has yet been established and so far no such tools exist for PI5P4Kγ. Moreover, a challenge facing any kinase inhibitor, the great majority of which interact with the ATP-binding site of their target, is for it to have both sufficient specificity (because all kinase ATP-binding sites show some structural similarity) and potency (cellular concentrations of ATP are in the millimolar range, so nanomolar affinity of an inhibitor is often required for micromolar efficacy in a cell). The high affinity and specificity of the phosphoinositide 3-kinase (PI3K)-δ inhibitor PIK-39 that results from a remarkable induced fit into the ATP-binding site of its target protein [17] is one example of an ATP-binding site competitor that overcomes these issues. A potential approach for increasing the kinase specificity is to look for ATP-allosteric modulators, although in some cases (e.g. [18]) there are discrepancies between cell-based and isolated kinase inhibitory assays, making difficult the finding of this kind of inhibitor.

Herein, we report the characterization and use of a PI5P4Kγ-specific inhibitor NIH-12848 (full designation NCGC00012848-02), which we show interacts not with the ATP-binding site but with the region where PI5P probably binds, including the activation loop. We use the inhibitor to begin the first exploration, in a kidney epithelial cell line, of the function of PI5P4Kγ. Also, we show how we can mutate PI5P4Kγ so that it becomes insensitive to NIH-12848, opening the possibility of chemical biology to explore the functions of all three PI5P4Ks.

## MATERIALS AND METHODS

### Enzyme preparation and mutagenesis

Recombinant enzyme was prepared essentially as described previously [12]. Protein from *PIP4K2C* (UniGene 6280511) or associated mutants, cloned into the expression vector pGEX6P (GE Healthcare) was expressed and purified from *Escherichia coli* BL21(DE3). Cultures were induced with 0.4 mM IPTG and probe-sonicated in the presence of protease inhibitors. GST fusion proteins of PI5P4Kγ and PI5P4Kγ+, a mutant with specific activity close to that of the active PI5P4Kα isoform [12], were harvested by binding to glutathione sepharose beads (GE Healthcare) and cleaved *in situ* with 50 units of PreScission protease (GE Healthcare) for 4 h at 4°C. Purity was confirmed by SDS/PAGE and protein concentration determined by colorimetric assay (Bio-Rad). Site-directed mutagenesis using the QuikChange technique (Agilent Technologies) was used to generate clones from which mutant forms of PI5P4Kγ and PI5P4Kγ+ were produced (for mutagenesis primers see Supplementary Table S1).

### Biochemical assays

Lipid kinase assays were performed as described previously [13]. Dipalmitoyl-PI5P (DiC16–PI5P) was purchased from Echelon Biosciences and after drying down in Eppendorf tubes was sonicated for 3 × 30 s in a Decon Ultrasonics sonicating bath. This lipid substrate (6 μM PI5P) and recombinant lipid kinase were added to the reaction mixture (200 μl of final volume) with 10 μCi [γ-^32^P]ATP and incubated at 30°C for 10–60 min. Lipids were extracted using an acidic phase-separation [19] and separated by 1D thin layer chromatography (2.8:4:1:0.6 chloroform:methanol:water:ammonia). Radiolabelled PtdIns(4,5)*P*_2_ spots were detected by autoradiography, extracted and counted with Ultima Gold XR scintillant (Packard) on a LS6500 scintillation counter (Beckman Coulter). Intrinsic ATPase activities of the enzymes were determined using the Transcreener ADP^2^ fluorescence polarization method (BellBrook Labs). A range of enzyme concentrations was assayed with ATP substrate (100 μM ATP, 60 min incubation at 22°C) and polarization units (mP) were read using a PHERAstar Plus microplate reader (BMG Labtech). Experimental values were interpolated from an ADP/ATP utilization standard curve and plotted using non-linear regression analysis with Prism 5.

### Hydrogen–deuterium exchange MS

Hydrogen–deuterium exchange (HDX)-MS experiments were performed according to previously published protocols [20,21]. In brief, HDX reactions were initiated in HDX buffer [20 mM HEPES, pH 7.5, 50 mM NaCl, 2 mM Tris carboxyl ethyl phosphine (TCEP)] by the addition of 98% ^2^H_2_O solution (10 mM HEPES pH 7.5, 50 mM NaCl, 2 mM TCEP), to give a final concentration of 78% ^2^H_2_O. Final protein concentrations were 1 μM. For all reactions with inhibitor, there was a 10-fold excess of inhibitor over protein and this was allowed to incubate for 30 min on ice before initiation of HDX. Four time points of exchange were carried out (3, 30 and 300 s at 23°C and 3 s at 0°C) and were terminated by the addition of a quench buffer (1 M guanidine-HCl, 0.8% formic acid), followed by snap freezing in liquid nitrogen. Samples were stored at −80°C until mass analysis.

#### Measurement of deuterium incorporation

Protein samples were rapidly thawed and injected on to a UPLC (ultra performance liquid chromatography) column immersed in ice, as previously described [21]. The protein was run over two immobilized pepsin columns in series (Applied Biosystems; porosyme) at 200 μl/min for 3 min and collected over a van-guard pre-column trap (Waters). The trap was subsequently eluted in line with an Acquity 1.7 μm particle, 100 x 1 mm^2^ C18 UPLC column (Waters), using a gradient of 5%–36% buffer B (buffer A 0.1% formic acid, buffer B 100% acetonitrile) over 20 min. Eluent from the column was injected on to a Xevo QTOF (Waters) acquiring over a mass range from 350 to 1500 *m/z*, with an ESI source operated at a temperature of 225°C and a spray voltage of 2.5 kV.

#### Protein digestion, peptide identification and mass analysis

Peptide identification was done by running tandem MS/MS experiments using a 3%–35% B gradient over 120 min with a Xevo QTOF (Waters). This was supplemented with a 20 min gradient separation to identify and correct the retention time for all samples. The MS tolerance was set to 3 ppm with a MS/MS tolerance at 0.1 Da. The resulting MS/MS datasets were analysed with the Mascot search within Mascot distiller (Matrix Science). All peptides with a Mascot score >15 were analysed using HD-Examiner Software (Sierra Analytics). The full list of peptides was then manually validated by searching a non-deuterated protein sample's MS scan to test for the correct *m/z* state and check for the presence of overlapping peptides. Ambiguously identified peptides were excluded from all subsequent analysis. The first round of analysis and identification were performed automatically by the HD-Examiner software, but all peptides (deuterated and non-deuterated) were manually verified at every state and time point for the correct charge state, *m/z* range, presence of overlapping peptides and the expected retention time. All HDX–MS results are presented as relative levels of deuterium incorporation and no correction for back exchange is applied, because no fully deuterated protein sample could be obtained.

### Cell culture

Immortalized mouse cortical collecting duct (mpkCCD) cells [22,23] were grown in defined medium [Dulbecco's Modified Eagle Medium (DMEM):Ham's F12 1:1 (v/v), 60 nM sodium selenate, 5 μg/ml transferrin, 2 mM glutamine, 50 nM dexamethasone, 1 nM tri-iodothyronine, 10 ng/ml epidermal growth factor, 5 μg/ml insulin, 20 mM D-glucose, 2% (v/v) fetal calf serum and 20 mM HEPES, pH 7.4] at 37°C in a 5% CO_2_, 95% air atmosphere. Medium was changed every 2 days and all studies were performed on cells that had been grown on plastic Petridishes or glass slides.

### RNAi

Depletion of specific PI5P4K isoforms was achieved by transfecting cells with siRNAs specific to each PI5P4K isoform. The siRNAs were obtained from Thermo Scientific Dharmacon (ON-TARGET*plus* SMART pool). Control siRNAs were sequences that would not be recognized by any part of the mouse genome. mpkCCD cells were plated at a density of 6 × 10^4^ per well (24-well/plate) 1 day before transfection, then were transfected with each siRNA at a final concentration of 25 pmol/ml using DharmaFECT 1 Transfection Reagent (Dharmacon). At 72-h post-transfection, a new round of transfection was performed and the cells were cultured for another 48 h.

### Morphological and immunocytochemical studies

Confluent cells grown on plastic Petridishes were examined under an inverted microscope (Zeiss) equipped with phase-contrast optics. Indirect immunofluorescence studies were performed on confluent cells grown on glass slides fixed with ice-cold methanol or 4% formaldehyde. Cells were processed for immunofluorescence using anti-Na^+^/K^+^-ATPase α-1 monoclonal antibody (Millipore) and anti-zona occludens-1 (anti-ZO-1) polyclonal antibodies (Millipore) and were examined under a Leica confocal microscope.

### Western blotting

Cells were grown to confluence, washed in cold PBS and lysed by scraping them off in boiling Laemmli sample buffer containing 20 mM 2-mercaptoethanol. Lysate was boiled for 5 min and viscosity was reduced by sonication using four bursts of 30 s each with 15 s on ice between each sonication step. Lysate was boiled as before and centrifuged at 20000 ***g*** for 5 min. The supernatant was run on 10% polyacrylamide gels, transferred to nitrocellulose membranes, blocked in TBS/0.05% Tween 20/5% non-fat dry milk and probed with antibodies diluted in the same solution. The following primary antibodies were used: polyclonal anti-PI5P4Kγ 1:2500 [9], monoclonal anti-α tubulin 1:5000 (Sigma–Aldrich). After washing in TBS/0.05% Tween and incubation with secondary antibody, protein was detected by Super Signal West Femto Maximum Sensitivity Substrate (Thermo Scientific).

### QT (quantitative)-PCR

Cellular RNA was extracted with Trizol reagent (Invitrogen) and 1 ng was reverse transcribed with Superscript III and random hexamers (Invitrogen). Quantification of gene expression of the three PI5P4K isoforms was assessed by using TaqMan Gene Expression Assays and TaqMan Gene Expression Master Mix (Life Technologies), using TATA-box-binding protein (TBP) as the reference gene.

## RESULTS AND DISCUSSION

### NIH-12848 is a specific PI5P4Kγ inhibitor

**Figure 1:**
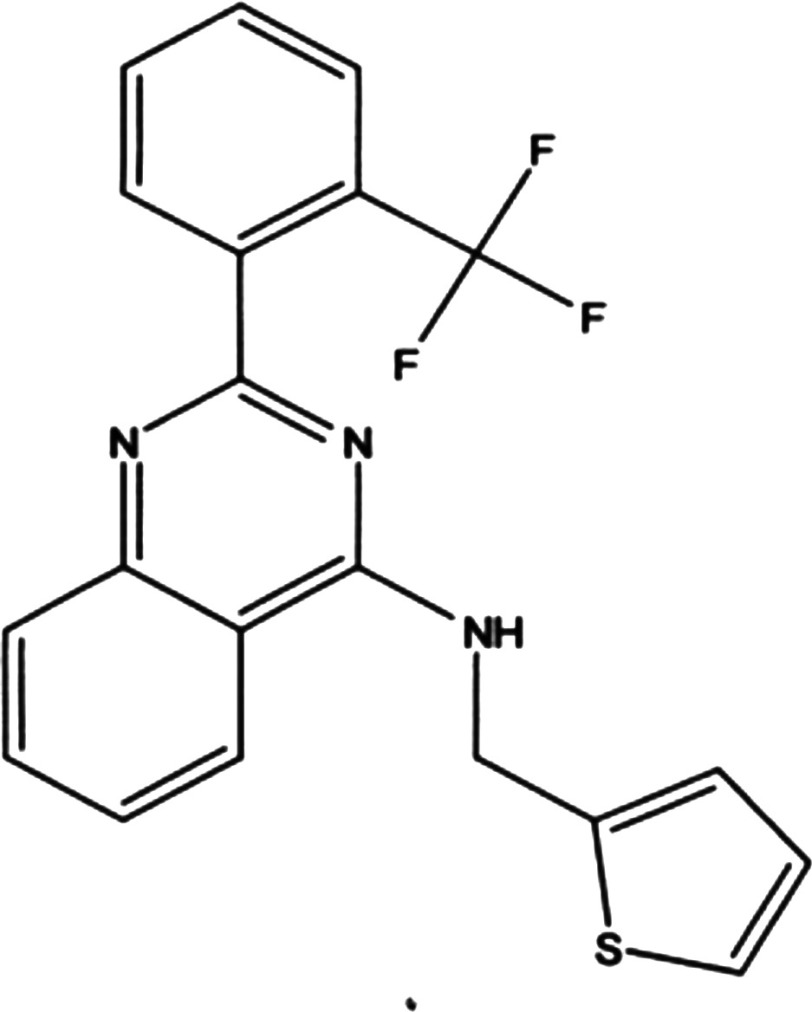
Structure of NIH-12848

**Figure 2:**
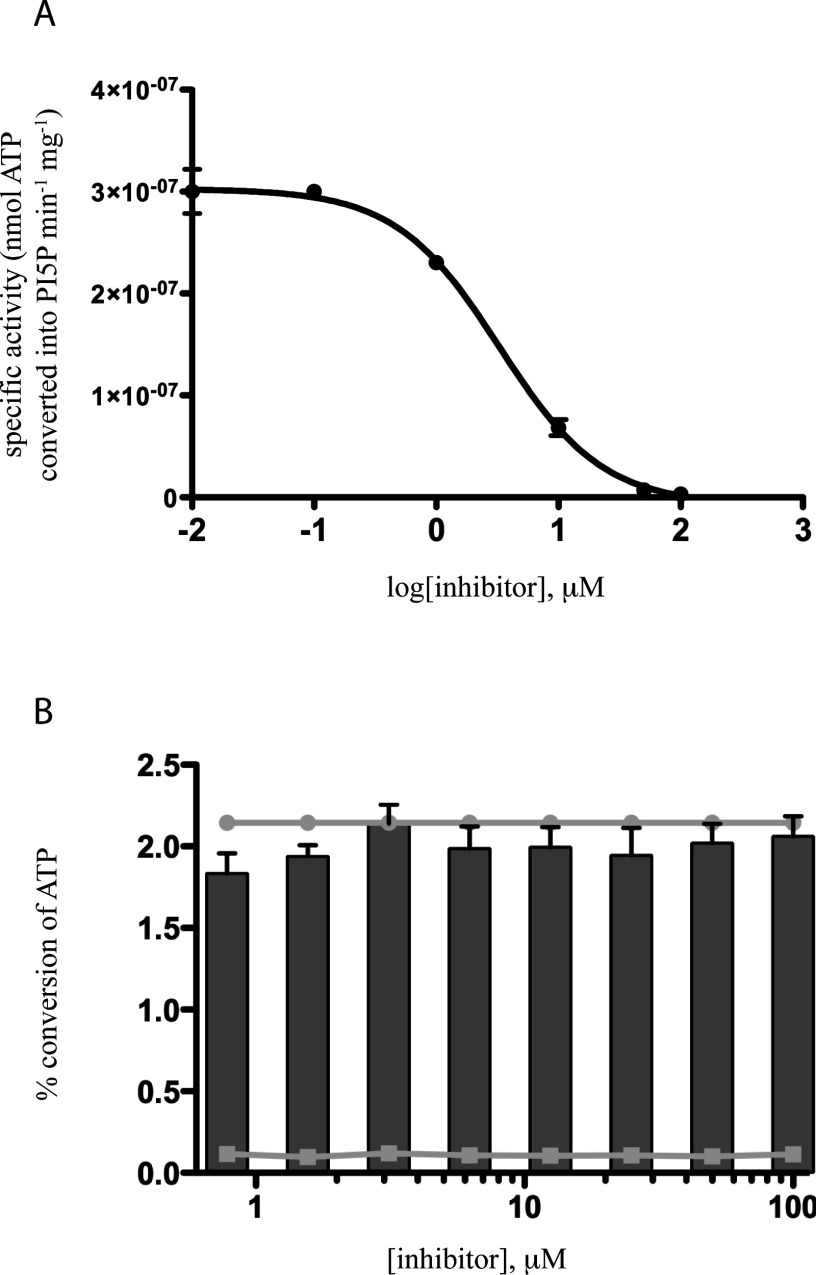
Effect of inhibitor NIH-12848 on PI5P4Kγ activity (**A**) Dose-response curve showing inhibition of PI5P4K activity over a range of inhibitor concentrations (0.01–100 μM). Points represent a mean of five replicates and error bars indicate ± S.E.M. (**B**) Effect of a range of inhibitor concentrations on intrinsic ATPase activity of PI5P4Kγ. Bars represent percentage ATP conversion by PI5P4Kγ (1 μM) in the presence of NIH-12848, in comparison with the indicated positive control (●; 1μM PI5P4Kγ in the absence of inhibitor). A negative control was also included (□; inhibitor only, no enzyme). *n*=3, error bars represent ± S.E.M.

NIH-12848 (Figure 1) initially emerged as ‘compound 1’ from a kinome profiling study, measuring its selectivity against a panel of 460 kinases (see Supplementary Figure S3 in ref [24]). As can be seen in the supporting information (Supplementary Table S2), NIH-12848 was exquisitely selective for PI5P4Kγ, with an apparent IC_50_ of 2–3 μM (Supplementary Figure S1). This screen did not include PI5P4Kα (Supplementary Figure S2) and to clarify the actions of NIH-12848 against PI5P4Ks we tested it in PI5P4K assays using PI5P and ^32^P-γ-ATP as substrates (see Materials and Methods). Under these conditions, PI5P4Kα was not inhibited by 100 μM NIH-12848 (Supplementary Figure S3A) and PI5P4Kβ showed a small but significant stimulation at the same concentration (Supplementary Figure S3B), whereas PI5P4Kγ was inhibited with an apparent IC_50_ of 3.3 μM (Figure 2), similar to the apparent *K*_d_ in the kinome profiling (above).

Because PI5P4Kγ has a very low catalytic activity [12], these assays use a final enzyme concentration of around 2.3 μM, so it is possible that NIH-12848 is actually more potent and we are, in part, titrating the enzyme protein. We therefore also tested NIH-12848 on nanomolar levels of the PI5P4Kγ+ mutant [12], which has a 300-fold higher activity than wild–type (WT) PI5P4Kγ and we found the IC_50_ of NIH-12848 to be about 1 μM (Supplementary Figure S3C). Below we show that the PI5P-binding site is the likely site of interaction for NIH-12848 and because the lipid PI5P does not have a precise concentration (not least this depends on its physicochemical structure), the number we deduce here can only be an empirical IC_50_, not an absolute *K*_i_.

### NIH-12848 interacts with the PI5P-binding site on PI5P4Kγ

Most of the mutations introduced into PI5P4Kγ to create PI5P4Kγ+ [12] involve the changing of residues that bind or stabilize ATP to the equivalent residues present in the much more active PI5P4Kα. So the marked contrast in sensitivities to NIH-12848 of PI5P4Kγ+ compared with PI5P4Kα suggested that NIH-12848 could be unusual among kinase inhibitors in that it might not be interacting with the ATP-binding site. This possibility was supported by an inability of NIH-12848 to inhibit the intrinsic ATPase activity of PI5P4Kγ (Figure 2B) and by the observation that including 100 μM ATP in assays using PI5P4Kγ+ (*K*_m_ for ATP, 2 μM [12]) had only a small effect on the ability of NIH-12848 to inhibit the enzyme near its IC_50_ concentration: 2 μM NIH-124848 inhibited PI5P4Kγ+ by 40±3.2% with carrier-free (approximately 16 nM) ATP and 64±2.8% with 100 μM ATP; the small shift in NIH-12848 sensitivity is probably an allosteric effect (see below).

**Figure 3:**
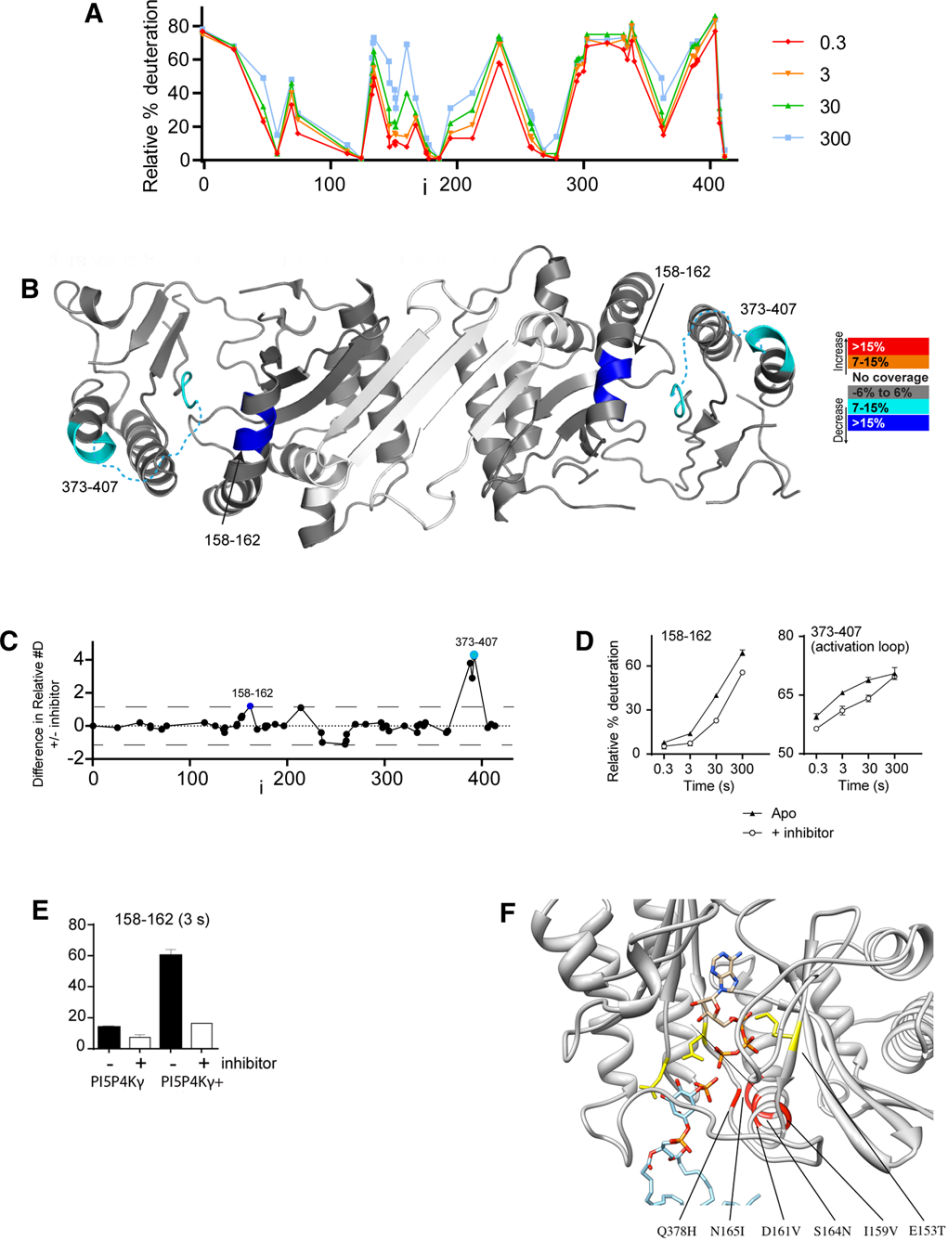
Binding of NIH-12848 to PI5P4Kγ studied by HDX–MS (**A**) Global HDX of Apo PI5P4Kγ. (**B**) Structure of PI5P4Kγ with changes induced by 10 μM NIH-12848 shown in pseudo-colour. (**C**) Summary of changes induced by NIH-12848. (**D**) Kinetics of HDX ± 10 μM NIH-12848 in two regions of PI5P4Kγ. (**E**) Quantification of HDX in region of residues 158–162 in PI5P4Kγ and PI5P4Kγ+ with or without NIH-12848. (**F**) Model of PI5P4Kγ active site (PDB 2GK9) with residues mutated in the present study highlighted in red (note that the E^153^T mutation engineered in PI5P4Kγ+ [12] is highlighted here in yellow, see text). The activation loop (containing Gln^378^) is not visible in the X-ray structure and is a conjectural conformation, as is the location of the ATP and PI5P substrates. Also in yellow (but not indicated) are Asp^374^, which is a conserved aspartate crucial for ATP binding [28] and Ala^386^, which dictates the substrate specificity of PI5P4Ks [26,27].

To determine directly the interaction site of NIH-12848 with PI5P4Kγ and PI5P4Kγ+, we turned to HDX–MS, a powerful tool for mapping protein conformational changes and binding epitope sites of interaction on proteins [21,25]. There are two peptides in PI5P4Kγ whose rate of HDX is altered significantly in the presence of NIH-12848 (Figures 3B–3D). One peptide spans residues 373–407 (Figures 3C and 3D), part of the enzyme's activation loop, which has been shown to govern the substrate specificity of PI5P4Ks and PI4P5Ks [26,27]. There was also a change in a peptide spanning residues 158–162 (Figures 3C and 3D), which is part of an α-helix that is near the activation loop in PI5P4Kγ (PDB 2GK9), designated α-helix 4 in the original annotated PI5P4Kβ structure [28] (Figures 3B and 3F). Intriguingly, in the PI5P4Kγ+ there was a significant increase in exchange in residues 158–162 compared with WT PI5P4Kγ and a correspondingly larger decrease in exchange upon addition of NIH-12848 (Figure 3E). These data point to the proposed PI5P-binding site of PI5P4Ks [26,27,28] as the likely area of NIH-12848 interaction.

### PI5P4Kγ can be made resistant to NIH-12848

Direct competition experiments (increasing PI5P and looking at the effect that this has on NIH-12848 potency *in vitro*) would be the most direct route to confirming the above suggestion. However, NIH-12848 is a hydrophobic compound, as is PI5P and it is likely that the two compounds would form mixed micellar structures when together in aqueous buffers, so any such ‘competition’ would be uninterpretable. To seek confirmation for the site of interaction of NIH-12848 we therefore undertook a series of mutational experiments, focusing on the regions of interaction suggested by the HDX–MS data. In the region 373–407 (see above), only Glu^378^ differs between PI4P5K isoforms and mutating this residue to the PI5P4Kα equivalent histidine made no detectable difference to the activity or NIH-12848 sensitivity of the enzyme (result not shown). In residues 158–162 (see above), Ile^159^ and Asp^161^ are both valines in PI5P4Ks α and β and mutating these together in PI5P4Kγ+ caused a small shift in sensitivity to NIH-12848 [inhibition by 10 μM NIH-12848 was 96% ± 1.5 in WT PI5P4Kγ+ and 71% ± 2.6 in the (E^378^H,I^159^V,D^161^V) PI5P4Kγ+ mutant].

Examining this region closely in the structure (Figure 3F) suggested three other residues that might interact with the substrate or inhibitor and which are different between PI5P4K γ compared with α/β. These are Ser^164^, Asn^165^ and Glu^153^, which are respectively asparagine, isoleucine and threonine in PI5P4Kα/β; of these, Glu^153^ has already been mutated to the PI5P4Kα/β-equivalent threonine in creating PI5P4Kγ+ [12]. We therefore introduced all five mutations of potential interest (Q^378^H, I^159^V, D^161^V, S^164^N, N^165^I) into PI5P4Kγ and PI5P4Kγ+ and both these mutant enzymes were completely resistant to NIH-12848 inhibition (Supplementary Figures S4A and S4B).

**Figure 4:**
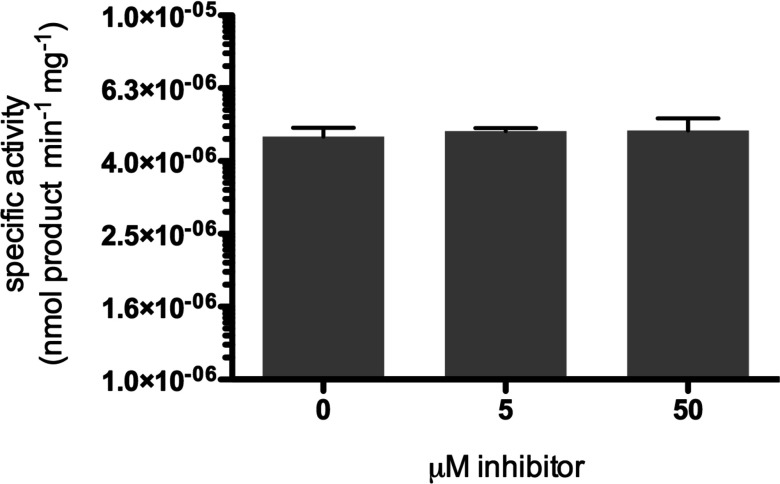
Effect of inhibitor NIH-12848 on PI5P4Kγ harbouring a single amino acid substitution PI5P4Kγ mutant N^165^I was assayed in the presence of 0, 5 and 50 μM NIH-12848 and showed no significant sensitivity towards this inhibitor. *n*=3–6 replicates for each condition, error bars represent ± S.E.M.

These data support the HDX–MS data suggesting that NIH-12848 is binding to this area of the PI5P4Kγ protein and they also imply that Ser^164^ and/or Asn^165^ are the major contributors to the sensitivity of PI5P4Kγ to NIH-12848. So we mutated only those two residues in PI5P4Kγ to their PI5P4Ks α/β homologues individually or together and found that whereas S^164^N PI5P4Kγ is as sensitive to NIH-12848 inhibition as WT PI5P4Kγ (Supplementary Figure S4C), N^165^I PI5P4Kγ is completely insensitive, with 50 μM NIH-12848 having no effect on this mutant (Figure 4). Note that the activity of N^165^I PI5P4Kγ was 6.7× higher than WT PI5P4Kγ in these assays and Supplementary Figure S4(D) summarizes the activities and sensitivities to NIH12848 of the mutants discussed in the present study.

Overall, these data show that Asn^165^ is the residue in PI5P4Kγ that is almost entirely responsible for its sensitivity to NIH-12848. There was no detectable change in the amide exchange rate at Asn^165^ when NIH-12848 binds to PI5P4Kγ or PI5P4Kγ+ (Figure 3) and the simplest interpretation is that NIH-12848 binds to the activation loop and α-helix 4 to induce a change in the structure in this region and that introducing an isoleucine (as in PI5P4Ks α and β) at residue 165 prevents this structural change from happening, perhaps because of a hydrophobic interaction with the activation loop (Figure 3F). Whatever the molecular mechanism, this residue could, in the future, be mutated in both alleles of the PI5P4Kγ gene in a cell line or a mouse to engender NIH-12848 resistance of the PI5P4Kγ, which would be a powerful chemical biology approach to eliminate completely any potential non-specific cellular effects of NIH-12848. Introducing the complementary mutations into PI5P4Ks α or β, possibly to make them NIH-12848-sensitive, could be an equally informative avenue of investigation.

### Effects of NIH-12848 on a kidney cell line

PI5P4Kγ is widely expressed in mammalian tissues, but is particularly high and is indeed the principal PI5P4K isoform in kidney [9]. It is expressed there in epithelial cells, particularly in the thick ascending limb and CCD, so to explore its cellular functions we chose mpkCCD cells, a cell line derived from epithelial cells in the cortical collecting duct of the kidney [22,23]. Western blotting confirmed an easily detectable level of expression of PI5P4Kγ in mpkCCD cells (Figure 6A). These cells grow to confluence and then form gap junctions between them. They then begin to form ‘domes’ in which they show a property of transporting epithelia when cultured *in vitro* on solid supports. ‘Domes’ are fluid-filled blisters formed between the solid growth surface and the cell layer and their formation is regarded as a sign of differentiated epithelia with active transport processes and with an intact epithelial barrier due to functional cell–cell contacts [22,23]. We found using quantitative PCR (QT-PCR) that while reaching confluence there is a three-fold increase in PI5P4Kγ mRNA, but no significant change in the two other PI5P4K isoforms (Table 1). We thought that PI5P4Kγ might be involved in epithelial cell polarity because in adult mammalian kidney PI5P4Kγ is present in intracellular vesicles with a distribution polarized towards the lumen [9] and in zebra fish (*Danio*) it is highly expressed in the pro-nephric duct throughout development (ZFIN Database: ZDB-IMAGE-030829-506).

**Table 1:** mRNA levels of PI5P4Ks in mpkCCD cells grown to confluence

Gene	*PI5P4Kγ*	*PI5P4Kα*	*PI5P4Kβ*
Polarized mpkCCD cells	3.19–3.46	0.99–1.01	0.51–0.53

The relative expression of the three genes *PI5P4Kγ*, α and β, with *TBP* as a reference gene, was determined in a reverse transcription quantitative PCR (RT-qPCR) assay. Values are expressed as fold difference in polarized cells (4 days after reaching confluence when domes are formed) relative to pre-confluent cells (no domes present).

**Figure 5:**
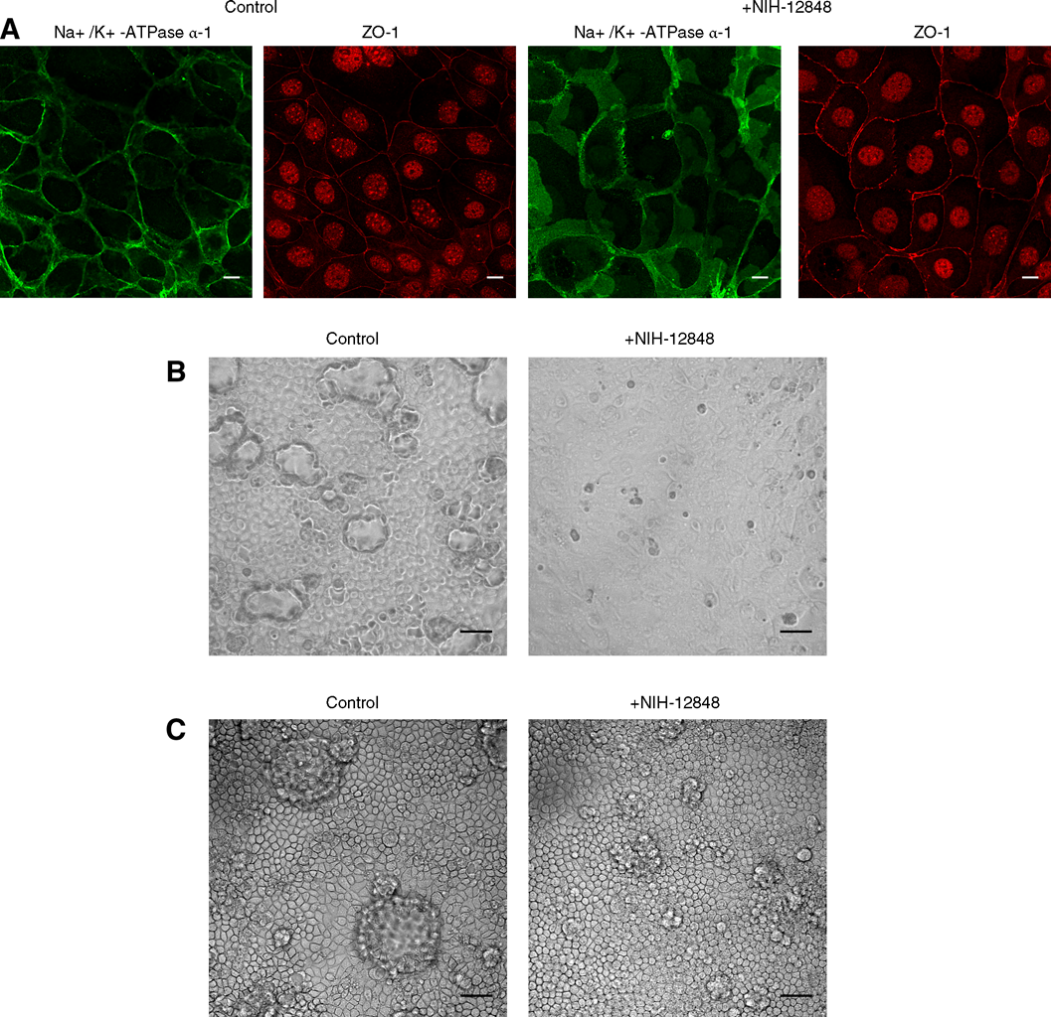
Effects of NIH-12848 on mpkCCD cells (**A**) Effect of 10 μM NIH-12848 on Na^+^/K^+^-ATPase α-1 localization. Cells were grown on glass coverslips at sub-confluence and then treated for 24 h with inhibitor or carrier (DMSO). They were stained for ZO-1 (gap junctions; red) or Na^+^/K^+^-ATPase α-1 (green) and viewed under a confocal microscope. Low resolution imaging showed that > 90% of the cells were as shown in these higher resolution images and at least three experiments were performed with identical results. Objective 100×; scale bars=10 μm. (**B**) Effect of 10 μM NIH-12848 on dome formation. Cells were grown on plastic dishes to confluence (no domes present) (left) and then for a further 48 h with inhibitor or carrier (right). Phase contrast images of cell monolayers are shown. Objective 20×; scale bars=50 μm. (**C**) Effect of 10 μM NIH-12848 after domes have formed. Cells were grown on plastic dishes for 5 days to allow dome formation (left) and then with inhibitor or carrier for a further 24 h (right). Phase contrast images of cell monolayers are shown. Objective 20×; scale bars=50 μm.

One easily monitored parameter is the redistribution of Na^+^/K^+^-ATPase from the cytoplasm to the basolateral plasma membrane that occurs when the cells reach confluence [29]. We explored the effect of 10 μM NIH-12848 (1 μM had similar but smaller effects) and found a complete inhibition of this redistribution, although the cells remained healthy and confluent, with gap junctions formed as revealed by immunocytochemistry of ZO-1 (Figure 5A). From this, we suggest PI5P4Kγ is involved in the progress towards a polarized epithelial phenotype.

Consistent with this possibility, the formation of ‘domes’ (see above) was also markedly inhibited (Figure 5B) by NIH-12848. Moreover, if NIH-12848 was added subsequent to dome formation, the domes disappeared with a detectable effect within 3 h that was maximal by 24 h (Figure 5B), indicating a dynamic relationship between PI5P4Kγ and the development and maintenance of this morphology. The molecular mechanisms underlying this relationship will require much further investigation, but the reported link between PI5P4Kγ and Rho [11], taken together with the well-known involvement of Rho in epithelial cell transport and polarity [30,31], offers one possibility for exploration.

### RNAi knockdown of PI5P4Kγ

**Figure 6:**
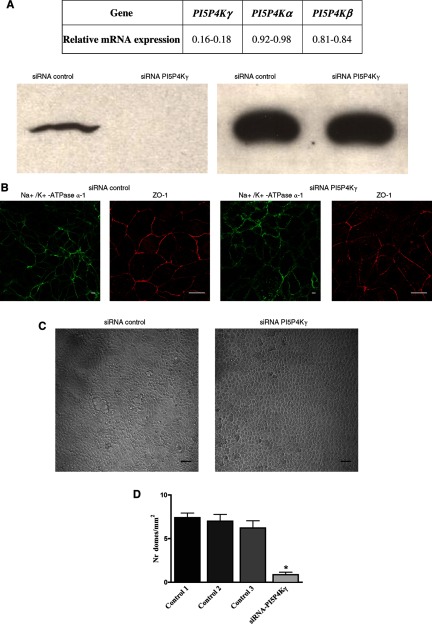
Effect of RNAi Knock-down of PI5P4Kγ or mpkCCD cells (**A**) Specificity and depletion efficiency of PI5P4Kγ siRNA. mpkCCD cells grown on plastic dishes were transfected with control siRNA or PI5P4Kγ-specific siRNA. After 72 h, the transfection was repeated. After a further 48 h, RNA was extracted and the expression levels of each PI5P4K isoform was evaluated by reverse transcription quantitative PCR (RT-qPCR). Values (above) are expressed as fold difference in knocked-down cells relative to control cells with *TBP* as the reference gene. Depletion of PI5P4Kγ was also evaluated in cell lysates by Western blot (below). Equal volumes of cell lysates were loaded on a SDS/PAGE (10% gel) and PI5P4Kγ was detected with an isoform-specific polyclonal antibody (see Materials and Methods). (**B**) Effect of siRNA knockdown of PI5P4Kγ on Na^+^/K^+^-ATPase α-1 localization. Cells grown on glass coverslips were depleted of endogenous PI5P4Kγ by siRNA as described in ‘Material and Methods’. Confocal sections of cells fixed and stained for Na^+^/K^+^-ATPase α-1 (green) and ZO-1 (red) are shown. These images are typical of two independent experiments and the images are representative of > 90% of cells examined. Objective 63×; scale bars=10 μm. (**C**) Effect of siRNA knockdown of PI5P4Kγ on dome formation. mpkCCD cells grown on plastic dishes were depleted of endogenous PI5P4Kγ by siRNA as described in ‘Material and Methods’. Phase contrast images of cell monolayers are shown. Objective 10×; scale bars=50 μm. (**D**) Surface density of cell domes was measured in mpkCCD confluent cell monolayers that were transfected with PI5P4Kγ-specific siRNA or only transfection reagent (control 2) or non-target oligos siRNA (control 3). In control 1, cells were not transfected. Values are expressed as means ± S.E.M. *P< 0.001 compared with control 3. Significant differences were analysed by Student's *t*test; Nr, number.

Our extensive molecular exploration of the interaction of NIH-12848 with PI5P4Kγ (above) emphasizes its remarkable specificity, but nevertheless it is possible that the effects we see are due to an off-target action. To gather some independent evidence that inhibition of PI5P4Kγ underlies the effects of NIH-12848 and thus to begin a validation of this inhibitor as a tool for exploring PI5P4Kγ function, we knocked down PI5P4Kγ in the mpkCCD cells by RNAi. Figure 6(A) and Supplementary Table S3 shows that the knockdown was specific and successful and Figures 6(B) and 6(C) show the effects of PI5P4Kγ knockdown on Na^+^/K^+^-ATPase re-localization (Figure 6B) and dome formation (Figure 6C). Within the limits of these analyses, both effects of NIH-12848 are mimicked by this knockdown.

Control experiments knocking down PI5P4Kα and β did not show the same effect on dome formation. PI5P4Kβ knockdown had no detectable effect (Supplementary Figure S5B), whereas knockdown of PI5P4Kα had a pronounced opposite (stimulatory) effect (Supplementary Figures S5A and S4B). We have no immediate explanation for this latter action, which we believe is likely to be independent of PI5P4Kγ activity because knockdown of PI5P4Kγ and PI5P4Kα together showed that PI5P4Kγ causes a similar and internally consistent inhibitory effect on the higher level of dome formation resulting from PI5P4Kα knockdown (Supplementary Figure S5C). Further studies, including using PI5P4Kα inhibitors (e.g. [15]) will be required to clarify this function of PI5P4Kα and its relationship with PI5P4Kγ. We have discussed elsewhere [12] the possibility that PI5P4Kγ may sometimes serve to target the more active PI5P4Kα to a specific cellular location.

Overall, these experiments yield the first functional insight into PI5P4Kγ's physiological role in epithelial cells. Much remains to be explored to clarify exactly what the enzyme is doing in this context, but the very similar effects induced in mpkCCD cells by inhibition with NIH-12848 and knockdown of PI5P4Kγ are a crucial first stage in a proof of principle of the potential usefulness of NIH-12848 in such an exploration. Our detailed mapping and manipulation of the molecular interaction of NIH-12848 with PI5P4Kγ also suggests ways to a wider use of this inhibitor in the exploration of functions of the PI5P4K family.

## Online data

Supplementary data

Table S2
