# Supplementary material for: The function of phosphatidylinositol 5-phosphate 4-kinase γ (PI5P4Kγ) explored using a specific inhibitor that targets the PI5P-binding site
Source: Biochem J. 2015 Feb 20;466(Pt 2):359–67. doi: 10.1042/BJ20141333 (PMC4687057; doi:10.1042/BJ20141333)
Supplement: Supplementary data [file bj4660359ntsadd.pdf]

Supplementary Tables

Supplementary Table 1.
Primer pairs that were used to generate amino acid substitutions in PI5P4Kγ and PI5P4Kγ+ mutants.

Amino acid change	Primer pair (forward/reverse)
Q378H	5'-TATCCTTACACATTATGATGCCAAGAAG<br>5'-TGGCATCATAATGTGTAAGGATATCAATG
I159V + D161E	5'-CCAGTGAGGACGTTGCTGAAATGCATAGCAAC<br>5'-GCTATGCATTTCAGCAACGTCCTCACTGGATACTG
S164N	5'-TGACATGCATAACAACCTCTCCAACTATCAC<br>5'-AGTTGGAGAGGTTGTTATGCATGTCAGCAATG
N165I	5'-TGACATGCATAGCATCCTCTCCAACTATCAC<br>5'-AGTTGGAGAGGATGCTATGCATGTCAGCAATG

Supplementary Table 2

Data from Kinome screen (see xls file)

Supplementary Table 3

mRNA levels of PI5P4Ks following RNAi induced knock down.

**Relative mRNA expression**	**PI5P4Kγ**	**PI5P4Kα**	**PI5P4Kβ**
**si-RNA-PI5P4Kγ**	0.16-0.18	0.92-0.98	0.81-0.84
**siRNA-PI5P4Kα**	1.10-1.40	0.15-0.20	1.00-1.10
**siRNA-PI5P4Kβ**	1.00-1.20	0.84-0.98	0.30-0.32

mpkCCD cells grown on plastic dishes were transfected with control siRNA or PI5P4K–specific siRNAs. After 72 hours, transfection was repeated. After further 48 hours, RNA was extracted and the expression levels of each PI5P4K isoform was evaluated by RT-qPCR.

Supplementary Figures

Supplementary Figure Legends

Figure S1
Dose-response of NIH-12848 on PI5P4Kγ in Kinome screen.

Figure S2
Data for PIP kinases extracted from Supplementary Table S2

Figure S3
Effect of inhibitor NIH-12848 on PI5P4K activity. Specific activities of PI5P4Kα (A) and PI5P4Kβ (B) are not inhibited when assayed in the presence of NIH-12848. N=3, error bars represent ±S.E.M. C) Dose response curve of PI5P4Kγ+ inhibited by NIH-12848. Values represent mean reduced activity compared to the mean of uninhibited PI5P4Kγ+ replicates from the same experiment.

Figure S4
Effect of inhibitor NIH-12848 on the activity of PI5P4Kγ mutants. A) Specific activity of PI5P4Kγ and B) PI5P4Kγ**+**, both with five amino acid substitutions (Q378H, I159V, D161E, S164N and N165I) were assayed in the presence of 0, 5 and 50 μM NIH-12848 inhibitor. C) Specific activity of PI5P4Kγ with a single amino acid substitution (S164N) in the presence of 1, 2 and 5 μM NIH-12848 inhibitor. D) Summary of the specific activities of a full range of PI5P4Kγ mutants in the presence of no (-) or 5 μM (+) NIH-12848. Substituted amino acids are listed below the relevant bars. WT = wild type PI5P4Kγ. N= a minimum of 3 replicates for each experiment, error bars represent S.E.M.

Figure S5
Effects of RNAi knockdown of PI5P4Kα and β on dome formation in mpkCCD cells.
A) siRNA for PI5P4Kα or control were applied in two rounds over 5 days. Typical images are shown.
B) Quantification of experiments typified by Fig S5A (with data for equivalent experiments with siRNA for PI5P4Kβ) Values are expressed as means ± SEM. *p<0.001. Significant differences were analyzed by Student's *t*-test.
C) Quantification of experiments in which PI5P4Kα and γ were knocked down alone or together. Values are expressed as means ± SEM. *p<0.001. Significant differences were analyzed by Student's *t*-test.

Supplementary Figures

Figure S1

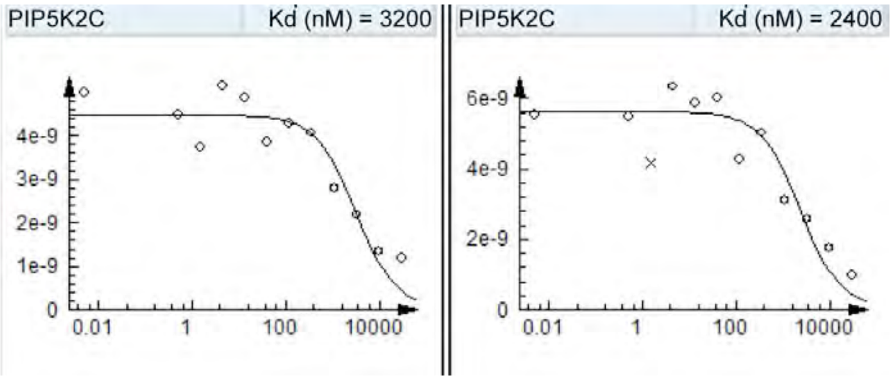

Figure S2

PIP5K1A	100
PIP5K1C	100
PIP5K2B	100
PIP5K2C	8.8

Figure S3

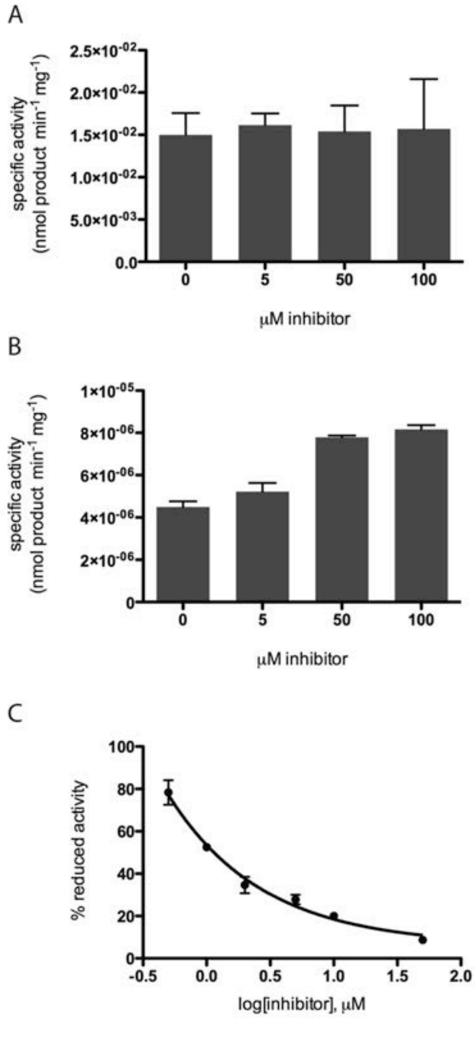
A
2.5×10-02
2.0×10-02
1.5×10-02
1.0×10-02
5.0×10-03
0.0
specific activity
(nmol product min-1 mg-1)
0
5
50
100
μM inhibitor
B
1×10-05
8×10-06
6×10-06
4×10-06
2×10-06
0
specific activity
(nmol product min-1 mg-1)
0
5
50
100
μM inhibitor
C
100
80
60
40
20
0
% reduced activity
-0.5
0.0
0.5
1.0
1.5
2.0
log[inhibitor], μM

Figure S4

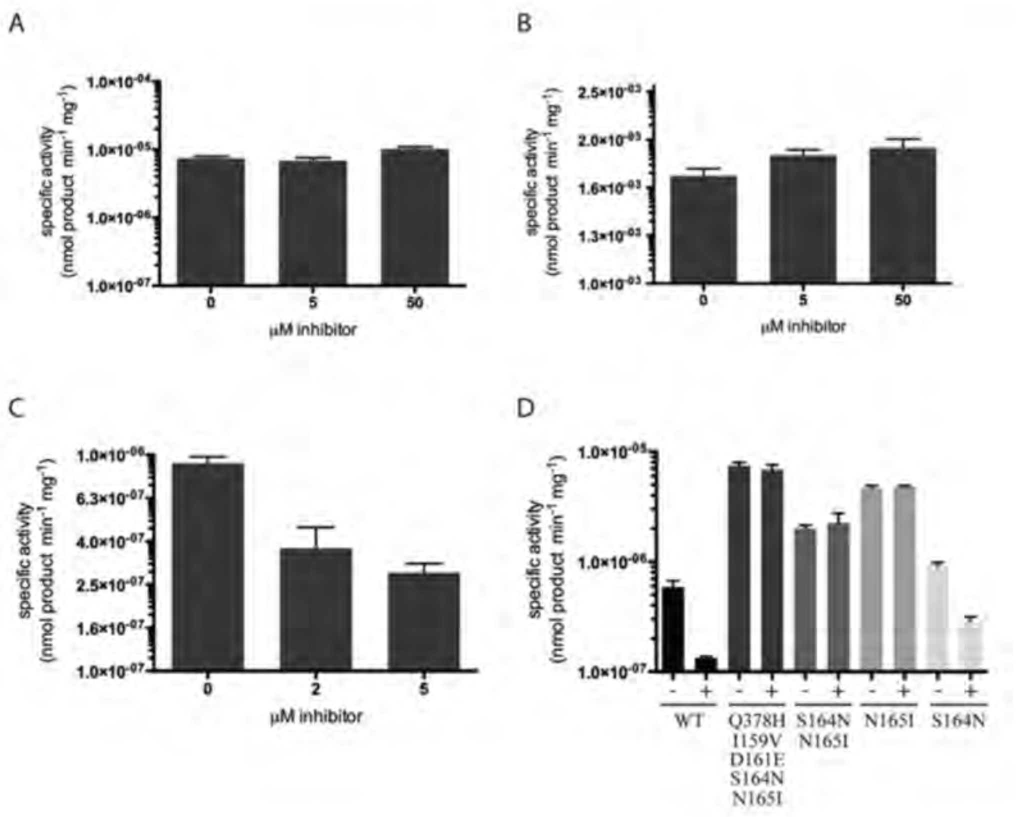
A
1.0×10-04
1.0×10-05
1.0×10-06
1.0×10-07
specific activity
(nmol product min-1 mg-1)
0
5
50
μM inhibitor
B
2.5×10-03
2.0×10-03
1.6×10-03
1.3×10-03
1.0×10-03
specific activity
(nmol product min-1 mg-1)
0
5
50
μM inhibitor
C
1.0×10-06
6.3×10-07
4.0×10-07
2.5×10-07
1.6×10-07
1.0×10-07
specific activity
(nmol product min-1 mg-1)
0
2
5
μM inhibitor
D
1.0×10-05
1.0×10-06
1.0×10-07
specific activity
(nmol product min-1 mg-1)
- + - + - + - + - +
WT
Q378H I159V D161E S164N N165I
S164N N165I
N165I
S164N

Figure S5

S5A

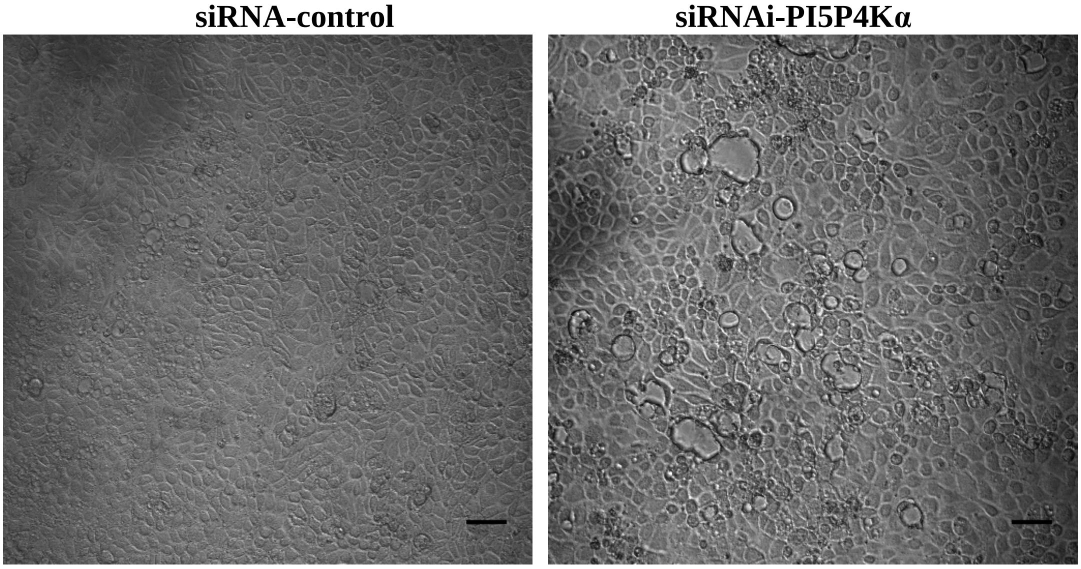

S5B

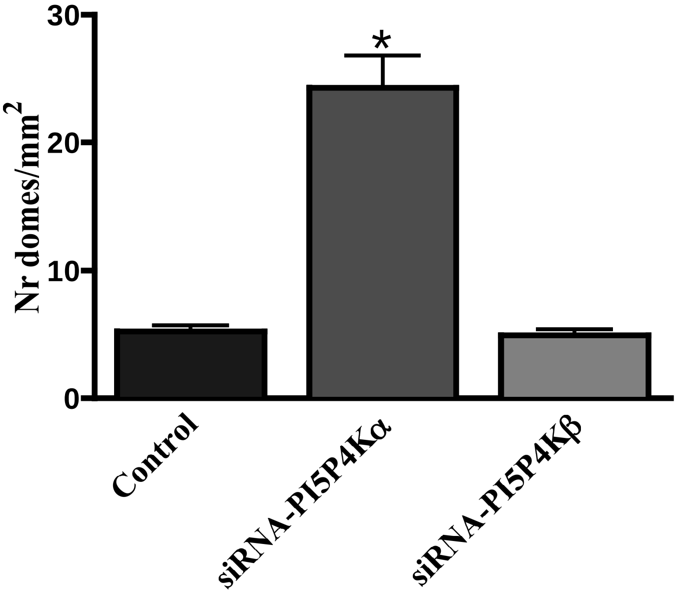

Fig S5C

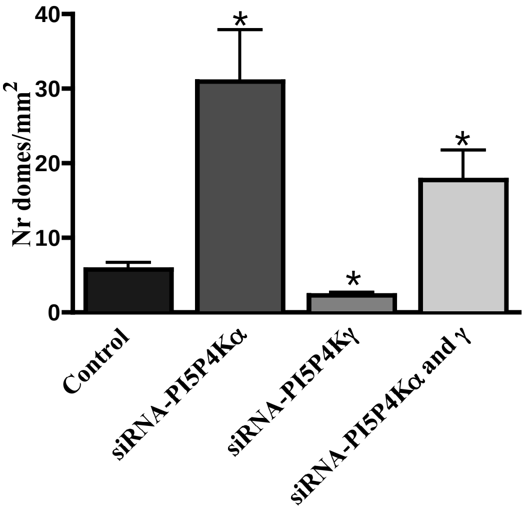

Nr domes/mm2
40
30
20
10
0
*
*
*
Control
siRNA-PI5P4Kα
siRNA-PI5P4Kγ
siRNA-PI5P4Kα and γ
